# Client Perspectives on Addressing Intimacy, Romance and Sexuality in Early Psychosis Intervention Programmes

**DOI:** 10.1111/eip.70014

**Published:** 2025-02-11

**Authors:** Stephanie M. Woolridge, Emma Wilkinson, Isabelle Hau, Chloe A. Stewart, Savie Edirisinghe, Robert Aidelbaum, Michael W. Best, Christopher R. Bowie

**Affiliations:** ^1^ Department of Psychology Queen's University Kingston (ON) Canada; ^2^ Department of Psychology University of Toronto Scarborough Scarborough (ON) Canada; ^3^ Department of Psychology The University of Guelph Guelph (ON) Canada; ^4^ Department of Psychology Toronto Metropolitan University Toronto (ON) Canada

**Keywords:** mental health recovery, patient care planning, psychotic disorders, schizophrenia, sexual health, social relationships, social support

## Abstract

**Objective:**

This study investigated the perspectives of clients in early psychosis intervention programmes regarding the types, quality and relevance of information they desire and receive, particularly related to intimacy, romance and sexuality.

**Methods:**

Participants (*N* = 35) rated the degree to which they desired and received information on topics related to treatment and recovery, as well as the quality and importance of that information.

**Results:**

Between 25% and 50% of participants desired information on friendships, sexual functioning, sexual desire, sexual risk factors and romantic relationships. Less than half of participants who wanted information on romantic relationships, friendships, sexual desire and sexual functioning received this information. When this information was provided, however, participants reported it to be of high quality.

**Conclusions:**

Findings demonstrate that intimacy, romance and sexuality remain areas of need that are relevant to recovery for people with psychosis, yet they continue to be insufficiently addressed in healthcare settings.

## 1 Introduction

Early psychosis intervention (EPI) programmes effectively improve outcomes and reduce relapse in psychosis (Catts et al. 2010; Malla et al. 2005; Marshall and Rathbone 2011). However, clients in EPI programmes have identified that the scope of these programmes neglects several areas that are important for their subjective recovery, namely: (1) intimate and/or romantic relationships and (2) sexuality and sexual functioning.

For many, intimacy, as an expression of humanity, is closely tied to perceptions of recovery and living a meaningful life. Yet, people with psychosis report that treatments addressing their personal relationships and sexual needs are unsatisfactory, particularly because of the pathologising of their sexual behaviour or devaluation of their sexual needs (Kelly and Conley 2004; Östman and Björkman 2013; Van Sant et al. 2012). In fact, in a sample of 1404 people with psychotic disorders, satisfaction with one's sex life was significantly lower than satisfaction in any other life domain, including mental health (Laxhman et al. 2017). Despite up to 90% of clients expressing needs related to sexual expression and intimacy, only 43% of clinicians recognised intimacy as a need for their patients, and only 10% recognised sexual expression (McCann 2010). Up to two‐thirds of psychiatrists do not enquire about sexual dysfunction, and only 17% feel competent assessing sexual dysfunction, despite 88% recognising its importance to their clients (Nnaji and Friedman 2008). Though intimacy and sexuality are integral to personal recovery for people with psychosis, these topics remain a peripheral focus in EPI programmes (Huggins et al. 2008; Quinn et al. 2011a; Raja and Azzoni 2003).

Clinician‐related barriers to addressing intimate and sexual needs include beliefs that these conversations are not appropriate or relevant, fears that conversations could lead to increased risk for clients and a discomfort or perceived lack of competency when talking about sex (Quinn et al. 2011a, 2011b; Southall and Combes 2020; Urry et al. 2019). Clinicians may view other issues (e.g., psychotic symptoms) as more pressing (Urry et al. 2019) and may inadvertently engage in strategies to silence discussions of sexuality (Huggins et al. 2008; Quinn et al. 2011a). The omission of these topics may contribute to interpersonal stigmatisation (Huguelet et al. 2015) and can create a diffusion of responsibility in healthcare that ultimately neglects clients' intimate or sexual concerns. These findings are in direct contrast to research with clients who desire to discuss issues related to sexuality and intimacy and who find these conversations constructive and informative (McCann et al. 2019).

To continue to improve outcomes in early psychosis, it is important that the interventions and resources being offered are consistent with clients' personal recovery goals. The present study sought to examine the preferences and experiences of clients in EPI programmes regarding the types, quality and relevance of information they desire and receive, particularly related to intimacy, romance and sexuality.

## 2 Materials and Methods

### 2.1 Participants and Procedure

Participants were outpatients with early psychosis recruited as part of a broader study examining associations between romance, sexuality, symptoms and social cognition in early psychosis. Participants had to be involved in an EPI programme for the first time within the past 5 years, read and speak English and be between the ages of 18 and 35. Study procedures were completed over Zoom either verbally or using Qualtrics survey software. Diagnoses were self‐reported and/or extracted from medical health records. The research received approval from the Queen's University Health Sciences and Affiliated Teaching Hospitals Research Ethics Board.

### 2.2 Measures

#### 2.2.1 Demographic Information

A demographics questionnaire collected information including age, self‐identified ethnicity, sex, gender, relationship and marital status, educational and occupational history and diagnostic history.

#### 2.2.2 Information From Healthcare Providers Survey

The information from Healthcare Providers survey (McInnis 2018) was adapted for the present study. Participants were shown 11 topics related to treatment and recovery in early psychosis (e.g., symptom management, return to work, social skills, romantic relationships, sexual functioning, etc.). For each topic, participants were asked several questions: (1) whether they wanted information on this topic; (2) if they received information; (3) the quality of the information and (4) relevance to their recovery. A percentage score (“Needs Met”) was calculated based on the number of participants who wanted and subsequently received information on the topic.

## 3 Results

### 3.1 Demographic Variables

Participants (*N* = 35) ranged in age from 20 to 30 years old (*M* = 24.26, SD = 3.32) with an average duration of EPI involvement of 27.76 months (SD = 16.91). Of the sample, 48.6% of participants identified as men, 40% as women, 8.6% as non‐binary and 2.9% as transgender men. With respect to race and ethnicity, 48.6% of participants identified as white, 20% as Asian, 11.4% as Black or Afro‐Caribbean, 11.4% as Indigenous, 5.7% as multiple ethnicities and 2.9% as Middle Eastern. Approximately half the sample was heterosexual (54.3%), and the majority of participants were single (77.1%). The sample was split in terms of whether participants had engaged in partnered sexual activity within the last year (40.0%) or had not (48.6%); 11.4% of participants declined to respond. Diagnoses included psychosis not otherwise specified (40%), schizophrenia or schizoaffective disorder (31.4%), mood disorder with psychotic features (22.9%), schizophreniform disorder (2.9%) and substance‐induced psychosis (2.9%). Comorbid diagnoses included anxiety disorders (34.3%), mood disorder (25.7%), personality disorders (25.7%), substance use disorders (25.7%) and post‐traumatic stress disorder (14.3%).

### 3.2 Information From Healthcare Providers

Across each topic, the number and percentage of participants who wanted and who received information on that topic are presented in Table 1. Less than half of participants who wanted information on social skills, romantic relationships, friendships, changes to sexual desire and sexual functioning received this information. Chi‐square analyses examined how the number of participants who desired and received information on each topic differed from symptom management, which is a primary goal of EPI programmes. Compared to symptom management, the proportion of participants who desired and received information about sexual functioning was significantly lower, and information about changes to sexual desire trended towards significance. Participants also rated the quality of the information they received and how important the topics were for their recovery; these data are presented in Table 1. Mean quality ratings were high or very high across all domains. Though medication and symptom categories were rated as most important for recovery, all mean domain ratings were in the range of ‘important’ (ratings of 1–3) rather than ‘unimportant’ (ratings of 4–6).

**TABLE 1:** Frequency of participants wanting and receiving information from healthcare providers.

	Wanted (%)	Received (%)	% Needs met	Versus symptom management^a^	*p*	Information quality *M* (SD)^b^	Importance for recovery *M* (SD)^c^
Medication management	24 (68.6)	24 (68.6)	100.00	*χ* ^2^ = 0.30	0.59	1.88 (0.61)	1.82 (1.27)
Mood symptoms	31 (88.6)	27 (77.1)	87.10	*χ* ^2^ = 0.042	0.84	2.11 (0.83)	1.72 (1.19)
Symptom management	31 (88.6)	25 (71.4)	80.65	—		2.21 (0.92)	1.75 (0.84)
Return to work/school	24 (68.6)	16 (45.7)	66.67	*χ* ^2^ = 0.21	0.65	2.47 (1.01)	2.03 (1.16)
Cognitive difficulties	29 (82.9)	17 (48.6)	58.62	*χ* ^2^ = 0.62	0.43	2.17 (0.92)	2.00 (1.16)
Sexual risk factors	11 (31.4)	6 (17.1)	54.54	*χ* ^2^ = 0.47	0.50	2.38 (1.60)	3.39 (1.67)
Social skills	27 (77.1)	11 (31.4)	40.74	*χ* ^2^ = 2.36	0.12	2.09 (0.70)	2.12 (1.09)
Romantic relationships	18 (51.4)	7 (20.0)	38.89	*χ* ^2^ = 2.00	0.16	2.43 (0.54)	2.88 (1.54)
Friendships	20 (57.1)	7 (20.0)	35.00	*χ* ^2^ = 2.69	0.10	1.86 (0.69)	2.45 (1.44)
Changes to sexual desire	13 (37.1)	3 (8.6)	23.08	*χ* ^2^ = 3.51	0.061	2.00 (0.00)	3.53 (1.63)
Sexual functioning	18 (51.4)	2 (5.7)	11.11	*χ* ^2^ = 7.72	**0.005**	1.67 (1.16)	3.09 (1.71)

*Note:* Bold indicates *p*‐value, the significance is exactly 0.005.

^a^ The percent of participants who had their needs met across each domain were compared to symptom management, a primary goal of EPI programmes.

^b^ Ratings of quality of information range from 1 (very high) to 5 (very low). Ratings were collected from participants who reported receiving information about each topic.

^c^ Ratings on importance range from 1 (very important) to 6 (very not important).

## 4 Discussion

This work examined participants' experiences seeking and receiving support in areas related to intimacy, romance and sexuality, finding that these topics remain areas of need that continue to be insufficiently addressed in healthcare settings. Aligned with previous work (McCann 2010; Nnaji and Friedman 2008), there was a considerable disparity between desires for information and the provision of this information; for example, as little as 11% of those who desired information on sexual dysfunction received that information. When this information was provided, however, participants reported it to be of high quality.

Consistent with past research, participants rated aspects of social, romantic and sexual functioning as valuable to recovery (McCann 2010; Huguelet et al. 2015; McCann et al. 2019; de Jager et al. 2018). The present findings encourage individualised treatment that is responsive to clients' subjective recovery goals. Mental healthcare that actively incorporates relationship development is likely to contribute to better short‐ and long‐term outcomes for persons with psychosis (Norman et al. 2005; Uzenoff et al. 2010). Normalising and validating difficult experiences with intimate relationships, as well as offering opportunities to discuss concerns about sexual health (e.g., addressing medication side effects, providing counselling for sexual issues) represent much‐needed contributions to social and overall well‐being.

Ultimately, sexual rights are based on freedom, dignity and equality. The highest attainable standard of healthcare is one that includes sexual health, sexual education and the possibility of pleasurable, satisfying and safe sexual experiences (International Planned Parenthood Federation 2008; Kismödi et al. 2017; World Health Organization 2006, 2010). Therefore, in addition to conversations around risk management (e.g., sexual risk behaviours, contraception/pregnancy, intimate partner violence), which are often prioritised (Southall and Combes 2020; Black et al. 2020; Ford et al. 2019; Urry et al. 2024), clinicians should aim to support clients in areas of healthy relationships and sexual pleasure to bolster overall well‐being, fulfilment and life satisfaction. A singular and (often) reactive focus on risk behaviours in the absence of other preventative conversations around healthy, pleasurable intimate relationships can have the unintended consequence of contributing to the pathologising of sexual behaviours among individuals with psychosis.

Given the interplay between mental health and intimate relationships, clinicians should be aware of relevant resources, referrals or interventions. Addressing these topics in healthcare settings is aligned with ethical practices and prevents these areas of life from ‘falling through the cracks’ due to a diffusion of responsibility across healthcare settings. Strategies for clinicians to address topics related to intimacy, romance and sexuality in healthcare settings include creating a space where clients feel comfortable discussing concerns, initiating conversations around these topics such that clients feel permitted to share their concerns if necessary (Annon 1976; Tuncer and Oskay 2022) and seeking additional training opportunities to build competency when navigating these topics (Berger‐Merom et al. 2022; Reissing and Giulio 2010).

This brief report should be considered alongside limitations. The data were collected via self‐report and the information provided may be limited in detail. Further, specific details about the providers of information (e.g., nurse, psychologist, social worker) were not collected; future research should collect this information to provide more specific recommendations for EPI programmes. Finally, the average duration of EPI involvement for participants was several years, and these findings may differ for individuals who have been recently admitted or who are in more acute stages of illness.

## 5 Conclusion

Recovery is understood to be an active, ongoing process of overcoming stigma and discrimination, participating in valued activities, feeling a sense of hope and personal meaning and engaging in important human relationships (Borg and Davidson 2008; Ridgway 2001). Person‐centred, recovery‐oriented care places the emphasis on a person's goals, wants and needs as drivers of the recovery process. The present findings emphasise the role of intimacy, romance and sexuality as both areas of need and aspects of the recovery process for many people. As such, EPI programmes are encouraged to implement flexible and responsive strategies to assist clients in accessing relevant information and resources to support their recovery goals related to intimate, romantic and sexual aspects of their lives.

## Conflicts of Interest

The authors declare no conflicts of interest.

## Data Availability

The data that support the findings of this study are available from the corresponding author upon reasonable request.
